# Introducing rapid diagnostic tests for malaria into registered drug shops in Uganda: lessons learned and policy implications

**DOI:** 10.1186/s12936-015-0979-6

**Published:** 2015-11-14

**Authors:** Anthony K. Mbonye, Sîan E. Clarke, Sham Lal, Clare I. Chandler, Eleanor Hutchinson, Kristian S. Hansen, Pascal Magnussen

**Affiliations:** Ministry of Health, Kampala and School of Public Health-College of Health Sciences, Makerere University, Box 7272, Kampala, Uganda; Department of Disease Control, London School of Hygiene and Tropical Medicine, Keppel Street, London, WC1E 7HT UK; Department of Global Health and Development, London School of Hygiene and Tropical Medicine, 15-17 Tavistock Place, London, WC1H 9SH UK; Department of Veterinary Disease and Biology, Centre for Medical Parasitology, University of Copenhagen, Copenhagen, Denmark

**Keywords:** Malaria, Rapid diagnostic tests, Drug shops, Policy, Lessons, Uganda

## Abstract

**Background:**

Malaria is a major public health problem in Uganda and the current policy recommends introduction of rapid diagnostic tests for malaria (RDTs) to facilitate effective case management. However, provision of RDTs in drug shops potentially raises a new set of issues, such as adherence to RDTs results, management of severe illnesses, referral of patients, and relationship with caretakers. The main objective of the study was to examine the impact of introducing RDTs in registered drug shops in Uganda and document lessons and policy implications for future scale-up of malaria control in the private health sector.

**Methods:**

A cluster-randomized trial introducing RDTs into registered drug shops was implemented in central Uganda from October 2010 to July 2012. An evaluation was undertaken to assess the impact and the processes involved with the introduction of RDTs into drug shops, the lessons learned and policy implications.

**Results:**

Introducing RDTs into drug shops was feasible. To scale-up this intervention however, drug shop practices need to be regulated since the registration process was not clear, supervision was inadequate and record keeping was poor. Although initially it was anticipated that introducing a new practice of record keeping would be cumbersome, but at evaluation this was not found to be a constraint. This presents an important lesson for introducing health management information system into drug shops. Involving stakeholders, especially the district health team, in the design was important for ownership and sustainability. The involvement of village health teams in community sensitization to the new malaria treatment and diagnosis policy was a success and this strategy is recommended for future interventions.

**Conclusion:**

Introducing RDTs into drug shops was feasible and it increased appropriate treatment of malaria with artemisinin-based combination therapy. It is anticipated that the lessons presented will help better implementation of similar interventions in the private sector.

## Background

Research has shown that formal and informal private sector providers are important suppliers of home-based treatment of illnesses [1, 2]. It has also been shown that in sub-Saharan Africa, 50 % of those with febrile illness access care through drug shops, and that 60 % of patients with febrile illness receive medicines from the private sector, including drug shops [1, 3]. Thus the private sector constitutes an important partner in the provision of healthcare for those with malaria in low-income countries [3–6].

For individuals infected with malaria, a key strategy to avoid serious illness and death is prompt treatment with an effective anti-malarial drug, as the majority of malaria deaths occur within 24 h of the onset of disease manifested by high fever [7]. In addition, current guidelines from World Health Organization (WHO) recommend that uncomplicated malaria should be treated with an artemisinin-based combination therapy (ACT) and that all patients suspected of malaria should have a parasitological test before treatment [7]. However, constraints to diagnosis and effective treatment, such as high costs in accessing health services and long distances to health units, have limited the implementation of the above recommendation for improving quality of care [8].

In 2010, the Affordable Medicine Facility malaria (AMFm), a donor subsidy at the ‘factory gate’ aiming to lower the cost of quality-assured ACT, was implemented. In addition to a price subsidy, the AMFm involved supportive interventions aimed at increasing access to ACT, including in-country branding and awareness campaigns for drug sellers and patients, training for ACT providers and greater access to malaria rapid diagnostic testing (RDT), [9]. An evaluation of AMFm showed that an ACT price subsidy quickly increased ACT availability and market share, and lowered consumer prices [10, 11].

Overuse of ACT as a result of over-diagnosis in the private sector is an additional concern where subsidies are used to improve the affordability of quality ACT to patients, through mechanisms such as AMFm [12]. Accordingly, WHO recommends universal access to parasitological diagnosis, encompassing all treatment providers, including the private sector [7].

The provision of RDT in drug shops potentially raises a new set of issues such as: implications of adhering to RDT results, management of severe illnesses, referral of patients, and relationship with caretakers. In addition, drug shops are unsupervised and have social relationships with the community which is substantially different from the interaction usually found in formal health service [13, 14].

A study to improve diagnosis and treatment of malaria, as well as referrals in registered drug shops was implemented in Uganda [15]. This paper presents lessons learned and policy implications for the benefit of policy makers who may be considering future large-scale implementation of malaria control in the private health sector.

## Methods

### Intervention

Between October 2010 and July 2012, a cluster-randomized trial introducing RDT in registered drug shops was implemented in 59 registered drug shops in a peri-urban area of Mukono District, Central Uganda. The design of the intervention was informed by formative research undertaken at baseline, described more fully elsewhere [16, 17]; the outcome of the intervention is also described elsewhere [15]. In brief, drug shops were randomized to diagnostic methods in two trial arms: those trained to use clinical signs and symptoms (the presumptive arm), and those trained to use RDT (the intervention arm) to diagnose malaria. Both arms were trained to recognize signs and symptoms of malaria, clinical signs for referral and how to make appropriate referrals to health facilities. The primary outcome was the proportion of febrile patients receiving appropriate treatment with ACT.

### Evaluation

An evaluation was undertaken between November 2011 and February 2012. The details of the methods have been published elsewhere [8, 15, 18]. Briefly, the quantitative evaluation was supplemented by a qualitative evaluation using focus group discussions (FGDs) which explored the intended and unintended effects of intervention for patients, drug shop vendors (DSVs) and other healthcare providers, including referral process and lessons learned.

### Ethical considerations

Ethical approval for the research was granted by review boards at the Uganda National Council of Science and Technology (Ref HS: 546) and London School of Hygiene and Tropical Medicine (No: 6049). Written informed consent was obtained from DSVs to participate in the trial, and from the patient (or caregiver) prior to household interviews; verbal consent was attained from patients attending shops. The study was registered with ClinicalTrials.gov on 2 September, 2010 (Trial registration: NCT01194557).

### Lessons learned

#### Design of the intervention

In order to design the key elements of the intervention, a stakeholder meeting was convened to share experiences with colleagues who had implemented such studies and were experienced in malaria control programmes, especially working with the private sector. Stakeholders included were Clinton Health Access Initiative, Malaria Consortium and The National Malaria Control Programme at the Ministry of Health [16].

An important lesson from the stakeholders’ meeting was to understand the dynamics in the drugs shops before implementing the intervention. Bringing together people with experience to help identify the key elements of the intervention was useful in understanding the dynamics. Similarly, the profit motive was identified as an important factor for the success of the intervention since ACT were very expensive on the open market (approximately US$3.00) and this could not be afforded by most of the public. The study decided to provide free ACT and RDT to DSVs and to conduct a willingness-to-pay study (WTP) to identify recommended prices that would ensure that majority of the public would purchase an RDT and a course of ACT [19]. This approach is recommended future studies.

Formative research studies were conceived to understand drugs shop operations and provide a baseline. Key deficiencies and challenges to appropriate diagnosis and treatment in drug shops, such as low knowledge of current treatment and diagnosis guideline, lack of training in national malaria treatment guideline, lack of reference materials (flow charts, job aids), limited record keeping in shops, and weak linkages with the public health system were identified during the formative research [17].

**Fig. 1:**
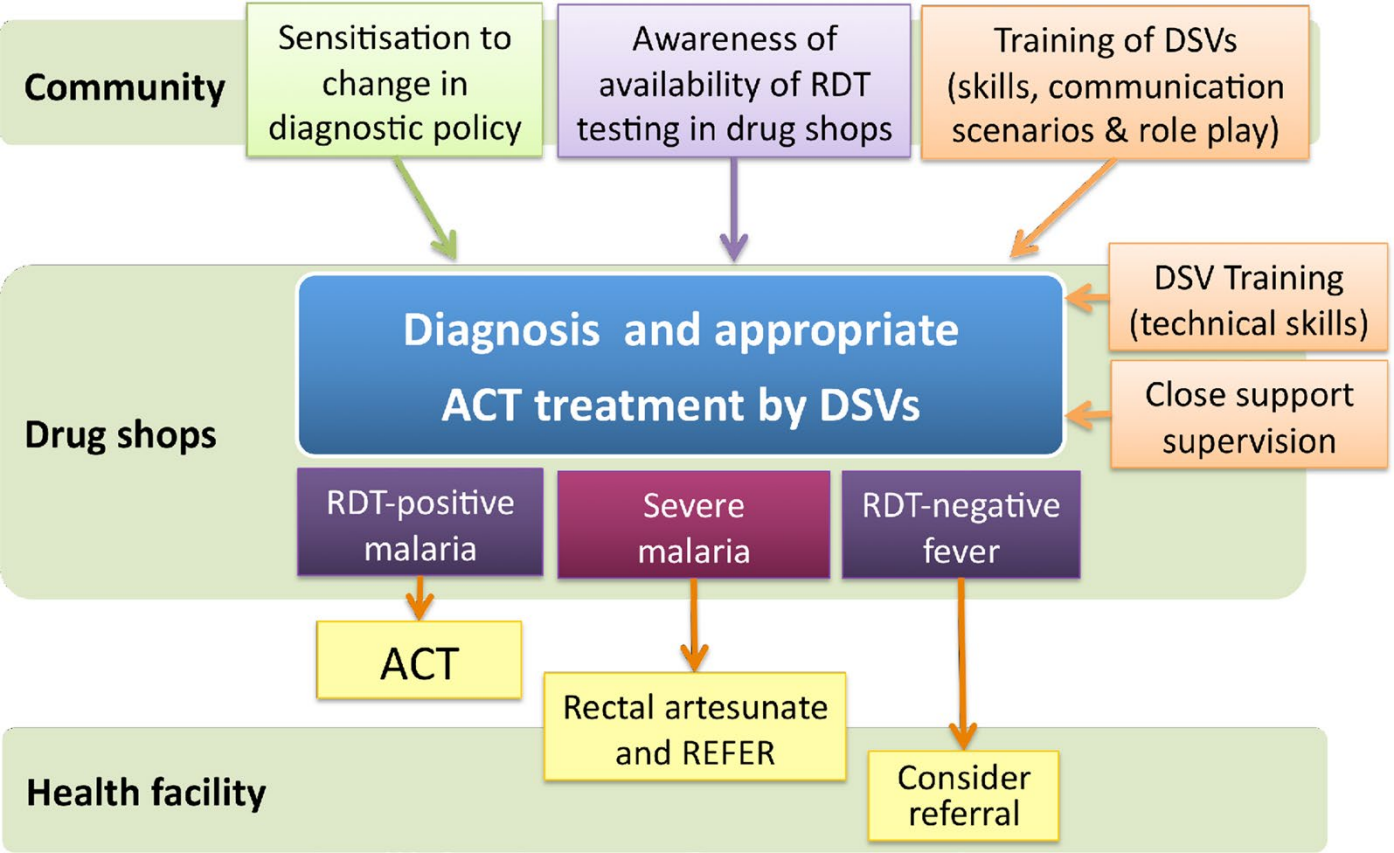
Intervention design and outcomes

An important lesson was how to balance real-life experiences of drug shop operations (baseline survey found out the level of training of DSVs, who actually runs the drug shops, opening hours, stock levels, knowledge on diagnosis, and malaria management) and to design appropriate supervision and evaluation modalities that would interfere minimally with DSVs’ behaviour (Hawthorne effect) [20], and also to reduce reporting workload. As part of the consenting process, DSVs were told that a sample of clients seen at their outlets would be followed up to assess their treatment-seeking behaviour after a visit to the drug shop, assess adherence to ACT treatment and the costs incurred. This was done to reassure DSVs that they were not being closely monitored, and to reduce the risk of suspicion and breach of trust between the research team and DSVs. Mystery drug shop visits were not part of the evaluation of this intervention [21]. A random sample of clients was followed up at home 4 and 14 days after their initial visit to a DSV. The key elements of the intervention are summarized in Fig. 1. The design of the intervention has been presented in detail elsewhere [16].

#### The involvement of the district health team and the community

Before implementation of the intervention, involvement of the district health team was considered necessary for three reasons: to enhance ownership and future sustainability of the research project; to draw upon their experiences regarding training and supervision of drug shops; and, to act as gate-openers to the communities. While designing the training curriculum and information materials, the district health educator and the District Assistant Drug Inspector (DADI) were involved.

It was considered important to create community awareness about the study using existing community structures at sub-county and parish levels as the new malaria treatment policy (to test all fever cases before treatment) was not widely disseminated. The village health team (VHT) trainers at sub-county level were trained and subsequently they trained VHTs in the study communities. The communities were sensitized to the current policy on malaria diagnosis and management (that not all fevers were malaria and all fevers had to be diagnosed by taking a blood sample to confirm malaria). The sensitization of communities was done through VHTs, local radios and churches. The lesson learned was that the involvement of the district in the design and implementation of the intervention and community sensitization by VHTs was important for the success of the intervention; and it is recommend for future studies and projects.

#### Implementing the intervention

Drug shops were invited to participate in the training; out of a total of 65 registered drug shops that consented to participate in the study, six drug shops failed to attend training because they thought the intervention would not work and that it would impact on their businesses by losing profits.

Close support supervision after training was necessary to ensure the drug shops mastered the new skills. Standard operating procedures (SOPs) and job aids were designed and distributed to help them ensure a high quality of the intervention. It was decided that the first 3 months would suffice for this purpose. An important lesson was that a lot of new skills and practices were required by DSVs that were different from their usual practice. For example, explaining to the clients about the study and their rights as research participants was a new practice and this posed challenges. A clinical audit discovered that a few drug shops were not following the guidelines, especially explaining patient rights. To address this, all the DSVs were retrained on patient rights and agreed to hold quarterly meetings with all DSVS to share experiences and learn from each other how to solve emerging problems. A number of DSVs were identified in each cluster to help in collecting forms and help supply of ACT and RDTs. Telephone contacts were established between the Principal Investigator, the field supervisor and DSVs to help whenever any drug shops experienced stock-outs. The details on how the intervention was implemented have been presented in detail elsewhere [16].

Data collection from DSVs was anticipated to be a challenge in two aspects: to get accurate data from DSVs, as record keeping was not usual practice in these outlets; to assess this aspect, ACT treatments we compared with records by the DSVs and the stock cards. Similarly, a sample of RDTs was re-tested to assess whether the results matched what the DSVs had recorded. It was found, after they were collected and re-read by the research team, RDTs could be interpreted accurately by DSVs. The lesson learned was that it was feasible to introduce routine record keeping into drug shops as part of the health management information system.

#### Evaluation of the intervention

The primary outcome results have been published elsewhere [15]. In brief, the proportion of febrile patients who received appropriate ACT treatment was 72.9 % in the intervention arm versus 33.7 % in the control arm, an increase of 36.1 % (95 % CI 21.3–50.9), p < 0·001. The majority of patients with fever in the intervention arm accepted to purchase an RDT (97.8 %), of whom 58.5 % tested RDT-positive. DSVs adhered to the RDT results, reducing over-treatment of malaria by 72.6 % (95 % CI 46.7–98.4), p < 0·001) compared to DSVs using presumptive diagnosis (control arm).These findings present robust evidence of scaling up RDTs in the private sector.

A random sample of 506 patients was followed at day 4 and 14 after the visit to the drug shop to evaluate the effect of the intervention on adherence to treatment, treatment-seeking practices after a drug shop visit, household costs, and referral practices. A major challenge was the time taken to follow up patients, locating their homes and the costs involved. In most cases researchers had to visit a home three times to get a response. A clinical evaluation of patient status would have been more informative. Patient reports of their health status were relied on. This has its own limitations because of variation in the understanding of health status between individuals. What is feasible on a routine basis is to ask patients who have not improved to come back for advice and possible referral.

From the inception of the study it was interesting to evaluate DSVs’ adherence to RDT results and how ACT prescriptions were recorded. There was good comparison between the two parameters. The DSVs’ records were compared with what treatment the patients in the sample were given. This yielded discrepancies in a few cases that were RDT-negative. This was because patients may not have known exactly what was prescribed. In future studies/interventions patients should be given a copy of a treatment form.

The referral practices after drug shop visits were evaluated and focussed on the following: what advice was given by DSVs, whether a referral form was provided, what the DSVs recorded as referral, and whether patients took up the referral advice. DSVs were viewed as offering a convenient and potentially cost-effective solution to patients where travel time and finances were important barriers to accessing the formal health system. The evaluation showed that introduction of RDTs at drug shops helped to improve the desirability of DSVs in the eyes of their patients [8, 18].

Despite the above, there was poor referral uptake. Fear of the implications of referral by DSVs, coupled with mistrust of RDT-negative results by both DSVs and patients led to poor referral uptake. Other constraints were the high costs of transport to the health facilities, and public health workers who were dismissive of the referral forms (in Luganda) coming from providers they distrusted. Further studies are needed to assess how the barriers identified in the present study could be addressed to improve referral.

### The policy implications

Based on a policy framework proposed by Walt and Gilson 1994 [22] that describes key elements (content, process, context, and actors) that influence policy development and implementation, we drew the following policy recommendations (summarized in Table 1).

**Table 1:** Summary of lessons and key policy recommendations

Lessons learned	Key policy recommendations
A workshop was held involving stakeholders with experience in the private sector to design key elements of the intervention	To scale-up RDTs and ACT requires the involvement of the districts, NMCP, NDA and development partners, e.g., Clinton Health Access Initiative (CHAI), Malaria Consortium, FIND, Uganda Health Marketing group (UHMG), and community-based civil society organizations
A baseline study noted poor regulation of drug shop practices	NDA should recruit more personnel to register drugs shops and support DADI in the supervision and regulation of drug shops. More personnel should be recruited at district level to assist DADI in supervision of drug shops
A baseline study documented poor record keeping in drug shops	Government to introduce DHIS2 to private health sector in order to address issues of data reporting coverage and to facilitate planning and management of health services
A baseline study noted poor quality of care at drug shops	Conditions should be attached to renewal of licences, mainly: minimum staff qualifications and evidence of continuing medical education
Drug shops were giving injections and antibiotics which are beyond their scope	NDA, DADI and professional associations to intensify regulation and supervision of drugs shops and other private health sector outlets to ensure patient safety
During evaluation of patient outcomes carewas taken not to influence DSV behaviour beyond what was realistic and sustainable	During scale-up RDTs and ACT, explaining the intervention and involving drug shops in the initial design processes generates acceptability and compliance
Community sensitization was an important factor in increasing access to mRDT and ACT at drug shops	Future interventions should use multi-media channels to deliver messages on key interventions. There is need to intensify messages on non-malaria fevers and care-seeking practices
There was poor referral uptake from drug shops because of mistrust between health facilities and drug shops	It is recommend that health workers be involved in training and supervision of the drug shops and in the design of interventions in the private sector
For drug shops to get involved in public health activities there have to be incentives	District health teams and stakeholders who involve drug shops in public health interventions should give DSVs certificates or trophies as signs of motivation

#### Content

The results of this trial showed that a package consisting of training DSVs on the new treatment and diagnosis policy, use of RDTs, community sensitization by VHTs, provision of subsidized RDTs and ACT, substantially increased appropriate treatment of malaria with ACT, directing ACT to patients who were parasite-positive (Table 2). This package is recommended for wide-scale implementation in the private sector.

**Table 2:** Summary of study outcomes

	Control clusters (n = 10)	Intervention clusters (n = 9)	
Clients with complete data	5797	7522	
Diagnosis and treatment	Frequency (%)	Frequency (%)	
Blood slide-positive	1841 (31.8)	3271 (43.5)	
Malaria treatment by infection status			
Blood slide-negative, received no ACT^1^	8 (0.2)	2662 (62.6)	
Blood slide-negative, received ACT	3948 (99.8)	1589 (37.4)	
Trial endpoints	Cluster mean (95 % CI)	Cluster mean (95 % CI)	
Proportion of blood slide-negative patients receiving ACT	99.8 (99.5−100.0)	42.4 (28.8−56.0)	p < 0.001
Proportion of febrile patients receiving appropriate treatment for malaria with ACT (95 % CI)	33.7 (25.8−41.5)	72.9 (67.3−78.6)	p < 0.001
Proportion of febrile patients seen within 24 h of onset of symptoms receiving appropriate treatment	26.8 (19.5−34.2)	52.8 (45.9−59.7)	p < 0.001
Proportion of febrile patients referred*	244 (3.6 %)	1019 (11.9 %)	p < 0.001
Reasons for referral			
Danger signs	105 (46.9 %)	667 (78.7 %)	p < 0.001
Other signs	119 (53.1 %)	180 (21.3 %)	
Recommendations from clients			
What do you recommend with regard to DSV?			
Improve health facilities of DSVs	16 (19.5 %)	19 (25.3)	
Improve geographical access	2 (2.4)	3 (4.0)	
Cheaper medication	8 (9.8)	12 (16.0)	
Greater stock and variety of medication	19 (23.2)	8 (10.7)	
Show patients test results RDT/blood samples	4 (4.9)	3 (4.0)	
Continue RDT use/expand to other DSVs	26 (31.7)	10 (13.3)	
Develop RDTs for other illnesses	11 (13.4)	20 (26.7)	
Which aspects of the treatment and advice you received at the drug shop were you happy with?			
Patient recovered/treatment worked	118 (54.4)	91 (41.5)	
Standard and form of healthcare provided	111 (51.1)	81 (37.0)	
Price of treatment and provision of credit	20 (9.2)	13 (5.9)	
Health facilities and stock of medication	9 (4.1)	2 (0.9)	
Offered referral and/or conditional advice	4 (1.8)	6 (2.7)	
Use of ‘blood test’/RDT	19 (8.8)	101 (46.1)	

* Within 24 hours of consultation

^1^ACT defined as either receiving artemether-lumefantrine of rectal artesunate

### Process

Record keeping was found to be poor in drug shops prior to the intervention. Several forms were introduced for evaluating the intervention in addition to several procedures. This was not a constraint to drug shops. Currently the Demographic Health Information System 2 (DHIS2) captures data from the public sector only [23]. Government should include the private health sector in the DHIS2, and this requires identifying key variables to be reported. This would improve coverage of data reporting and use of local data in planning and management of health services. Government would have to invest in this strategy. As a condition to renewal of licences, drug shops should sign to this reporting. Training and supervision should be the incentives.

Although the impact of community sensitization and the involvement of VHTs were not evaluated, it is believe community sensitization was important in increasing access of RDT and ACT at drug shops. Future interventions could use multi-media channels (such as social media) to drive home messages on interventions. Messages of policy changes, such as diagnosis of fever before treatment, helped to inform the community and they appreciated the RDTs, except in few cases where the negative tests were not trusted in the face of patients having fever. There is also a need to pilot schemes that link communities to the health system, such as strengthened referral of patients.

#### Actors

The regulation of drug shops is a policy issue that government should address. In this study it was found out that drug shops were registered by the National Drug Authority (NDA) through the DADI. Some drug shops had a receipt indicating that they had paid the DADI and others had licences from the NDA. It is recommended that NDA recruit more personnel to register drugs shops and support the DADI in the supervision and regulation of drug shops. Similarly, more personnel should be recruited at district level to assist the DADI in regular supervision of drug shops. Action should be taken against drug shops not following regulations.

At baseline, it was found that some drug shops were giving injections while others stocked drugs, such as antibiotics which were beyond their capacity [24]. Although this is due to poor regulation, it has implications for patient safety [25]. It is recommended that district health teams and professional associations get involved in the regulation of drugs shops and other private health sector outlets to ensure patient safety.

#### Context

The scale-up of RDT and ACT requires government leadership to address issues of logistical supply of ACT and RDTs, price regulation and monitoring quality of these supplies. Under the AMFm arrangement it was thought that subsided ACT and RDT would be procured and distributed through identified pharmacies. Communities were supposed to be sensitized about prices and the colour label (green) of ACT. As a policy issue government could make an application to the Global Fund and subsidize ACT and RDTs in the private sector to increase access and equity, especially in rural areas.

Referral practices were assessed; and the major issue was the apparent mistrust between health facility staff and drug shops. To address this mistrust it is recommend that health workers be involved in training and supervision of DSVs. This could take place at health facilities to initiate a process where DSVs work closely with health workers and gain confidence in each other. District health teams could initiate this interaction. Discussions on how to improve referral, the type of referral forms, and how to handle emergency cases and non-malaria fevers could be discussed during these interactions. A pilot scheme to evaluate effectiveness of different approaches is recommended.

There is a need to identify incentives for drug shops to participate in public health interventions. For example, in Ghana the majority of chemical sellers expected some form of subsidy from the government for contributing to public health activities, government to supply them with vaccines, to have tax exemptions, and to be supplied with free drugs. In addition, they wanted their staff to be trained by the Ministry of Health, wanted scholarships for further training and staff to be seconded to their facilities [26]. Further studies are needed in Uganda and elsewhere to assess how incentives could impact on drug shop practices.

For drug shops to get involved in public health activities there have to be incentives. Certificates provided at the end of training were a sign of recognition that drug shops had been endorsed by the Government [18]. This was an incentive for drug shops to participate in the study, complete study forms and adhere to guidelines. It is recommend that the district health teams involve drug shops and other private health sector outlets in public health interventions and give them certificates or trophies as signs of motivation, recognition and approval.

## Conclusion

Introducing RDTs into drug shops was feasible and it increased appropriate treatment of malaria among febrile patents. Successes and challenges in designing, implementing and evaluating the intervention have been presented. Wide-scale implementation of RDTs in the private health sector based on these lessons is recommended.

## Abbreviations

RDTs: rapid diagnostic tests for malaria
ACT: artemisinin-based combination therapy
WHO: World Health Organization
AMFm: Affordable Medicine Facility malaria
FGDs: focus group discussions
NMCP: National Malaria Control Programme
MOH: Ministry of Health
DSVs: drug shop vendors
WTP: willingness-to-pay
SOPs: standard operating procedures
DADI: District Assistant Drug Inspector
DHIS2: Demographic Health Information System 2
VHT: village health team
NDA: National Drug Authority
